# Dependence of the Contact Resistance on the Design of Stranded Conductors

**DOI:** 10.3390/s140813925

**Published:** 2014-07-30

**Authors:** Youcef Zeroukhi, Ewa Napieralska-Juszczak, Guillaume Vega, Krzysztof Komeza, Fabrice Morganti, Slawomir Wiak

**Affiliations:** 1 Lille Nord de France, F-59000 Lille, France; E-Mails: youcef.zeroukhi@nexans.com (Y.Z.); fabrice.morganti@univ-artois.fr (F.M.); 2 LSEE, UA, Technoparc Futura, F-62400 Bethune, France; 3 Institute of Mechatronics and Information Systems Technical University of Lodz, Stefanowskiego 18/22, Lodz, Poland; E-Mails: komeza@p.lodz.pl (K.K.); swiak@wp.pl (S.W.); 4 Nexans, 62300 Lens, Bd de Marais, France; E-Mail: guillaume.vega@nexans.com

**Keywords:** stranded conductors, contact resistance, cable design, cable simulation, contact resistance measurement

## Abstract

During the manufacturing process multi-strand conductors are subject to compressive force and rotation moments. The current distribution in the multi-strand conductors is not uniform and is controlled by the transverse resistivity. This is mainly determined by the contact resistance at the strand crossovers and inter-strand contact resistance. The surface layer properties, and in particular the crystalline structure and degree of oxidation, are key parameters in determining the transverse resistivity. The experimental set-ups made it possible to find the dependence of contact resistivity as a function of continuous working stresses and cable design. A study based on measurements and numerical simulation is made to identify the contact resistivity functions.

## 1. Introduction

In the context of high metal prices—particularly of copper—the main material used in electrical conductors, a reduction in the use of raw materials is one of the challenges facing the cable industry today. Thus the usage of copper or aluminium (or any other conducting material) in the manufacturing of cables must be analysed to achieve the desired electrical conductance under the imposed conditions.

This paper presents partial results of our work concerning the development of a finite element model for three-dimensional modelling and design optimization of multi-strand conductors. One of the points hindering this study has been the lack of understanding in the area of contact between wires. Indeed, these areas of contact between the wires of the cable, are an essential problem for a model incorporating robust mechanical and electrical coupling. The characteristics of the contact electrical parameters between the conductor strands *versus* mechanical stress and the relative position of individual wires have a significant influence on the distribution of current lines in the cable [1–3]. This paper is focused on the characterization of the electrical contact resistance by experimental measurements and electromechanical simulation based on the finite element method. The authors' interest mainly focuses on the study of electrical Cu/Cu contact between two conductors under the influence of a compressive force. The attention is particularly concentrated on the study of the influence of the contact force and of the crossing angle between the wires, and the shape and the size of the contact area.

In the first phase, the experimental measurement device was prepared. First the bench for applying compression and measurement of the contact force was built. It also has a system for fixing the angle of intersection of the conductors. Measurements have been conducted for DC loads. The electrical contact resistance, for its part, is measured using a 4-pointmethod (Kelvin method) with amicro-ohmmeter. The measurement protocol and the experimental test conditions are specified.

In a second step, the results of several dozen tests are presented. The analysis of these results is performed in order to understand the influence and quantify the impact of each factor studied on the electrical behaviour of the contact interface, including its resistance. After each measurement the sample was examined under a microscope to first investigate the shape and the size of the imprint, and second to investigate the internal structure of the conductor.

The copper wires from which the measured samples have been made are produced using a process called cold deformation. The stranding and compacting process is a complex operation that combines two simultaneous displacements: translation and rotation. From these combined displacements, three distinct forces will act on the wires: traction, torsion and compression. To compare the measurement and simulation results, this process was modelled using the Abaqus program [4] which allows simulating the stranding and compacting operation to calculate the mechanical deformations, the residual stresses, the inter-strands contact pressure and the resultant plastic strain. This program detects the contact zone as well as calculates the stress distribution.

Thanks to the measured characteristics of the resistivity as a function of stress it is possible to takes into consideration the non-homogenous resistivity of the contact zone. The non-homogenous resistivity will be implemented and taken into account in the finite elements model by defining an electromechanical coupling strategy. This will allow us to perform a more detailed analysis of the electrical phenomena and their impact on the total electrical resistance of the conductive core.

Finally, the identification of the electrical contact resistance and resistivity of material according to acting stress were applied for simulation of the 1+6 cable. The simulation results for different samples are analysed and compared with experimental measurements to validate the numerical model.

## 2. Experimental Section

### 2.1. Method of Measuring of the Contact Resistance

There are many unknowns relevant to optimization of the electrical contact resistance and experimental techniques to measure the resistance and its dependence on various parameters, such as temperature, pressure and the contact angle [5–9]. The two main questions requiring an answer concern the value of the contact resistance between the strands of a multi-strand conductor at a given force, created by the stranding process or due to subsequent application of compression, and ways to reduce contact resistance by considering the shape, size and distribution of contact interface sand current paths.

**Figure 1.**
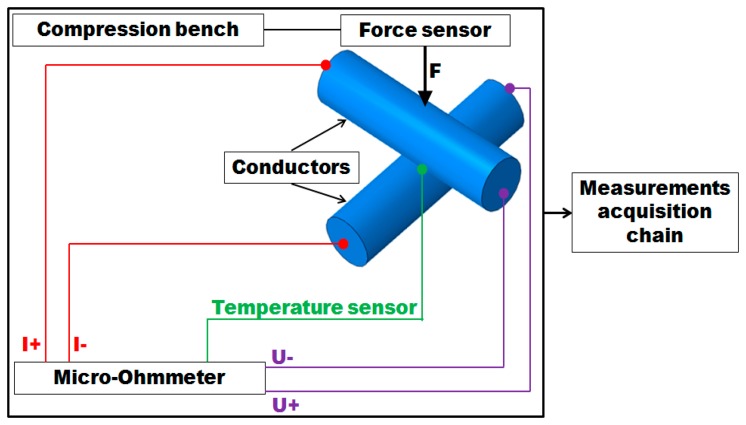
Diagram of the experimental measurement.

It is necessary to use the theory of mechanical contact in the analysis of the electrical contact. For this purpose a dedicated experimental device has been developed (“experimental bench”). This device is used to evaluate the electrical contact resistance between two conductors under the action of a force of pressure on the contact area of the cables. The contact surface between two solid conductors contains a number of mechanical contact blocks due to the inevitable surface roughness and lay length. The number and size of the electrical contact depends on many parameters like the force, the macroscopic form of the surfaces, the microscopic topology of the surfaces (roughness), the mechanical properties of materials (hardness, Young's modulus) and the geometry. We need to measure the electrical resistance of a contact established between two metallic conductors arranged in a precise angle beam, subjected to a force of progressive compression. The electrical contact resistance is measured according to the “4-points method” (Kelvin method).The overall diagram of the experimental measurement is shown in Figure 1.

**Figure 2.**
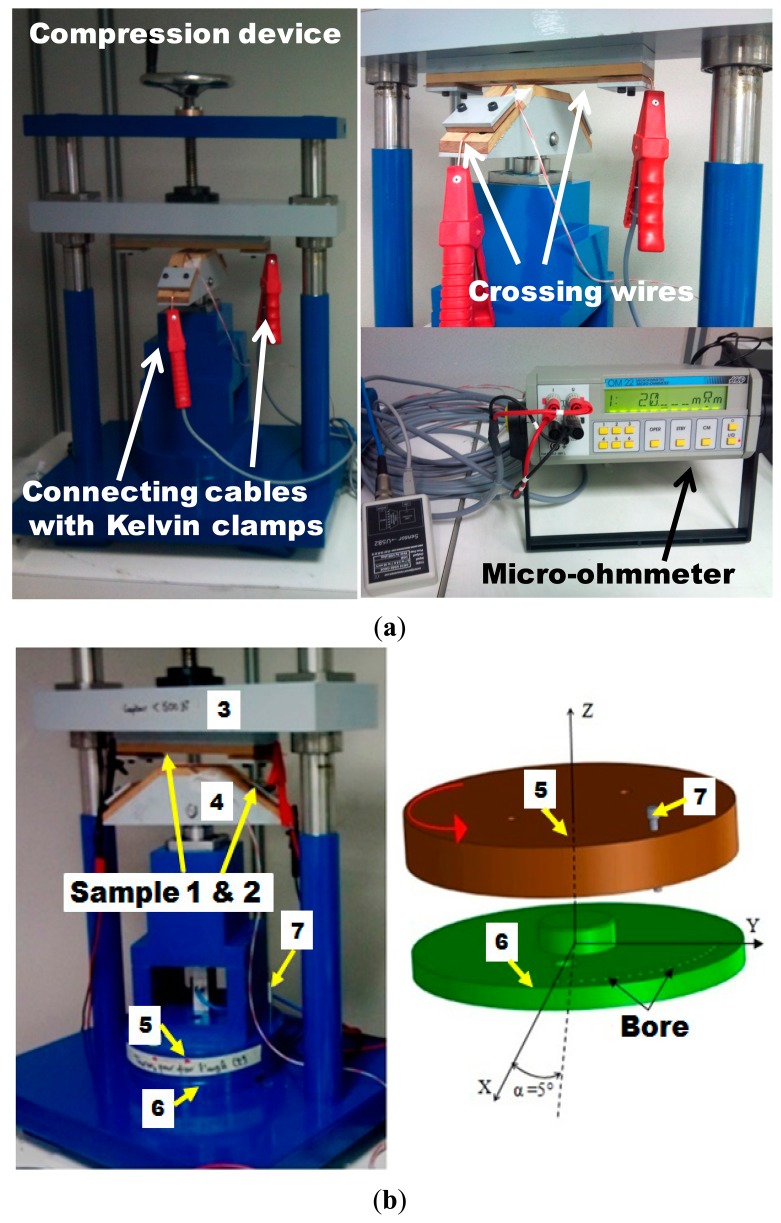

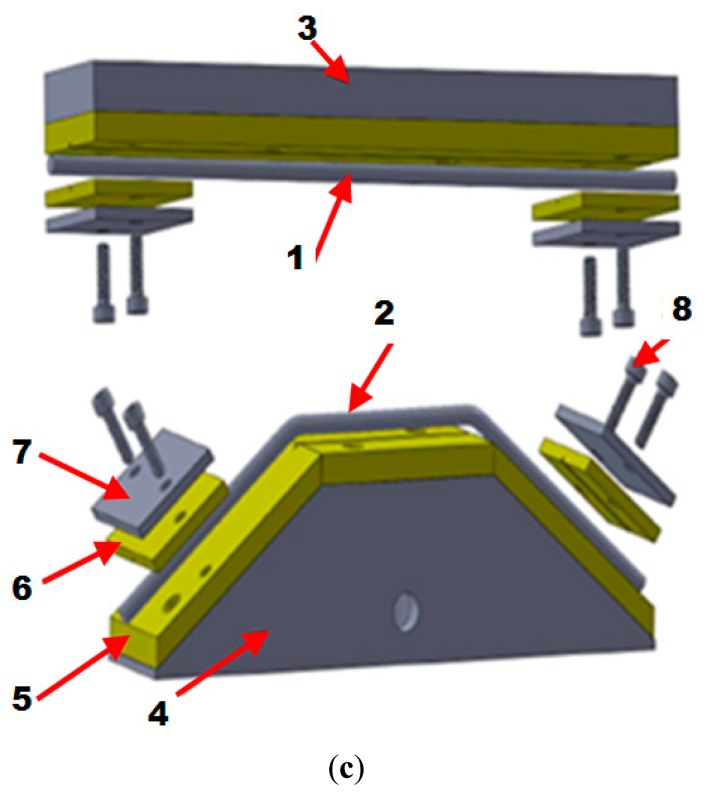
(**a**) The measuring bench; (**b**) Two disks allowing the adjustment of the crossing angle; (**c**) The system for fixing the wire.

Two elements are linked together: a mechanical part that works as a compression machine, and a second part for electrical measurements using the Kelvin method to measure the resultant contact resistance. The bench has a rotary plate to allow the measurement of the contact resistance at a variable angle between the crossing wires (Figure 2b). It has the possibility to set different crossing angles corresponding to the desired lay length of the stranded conductor. The contact resistance is measured using the 4-points method: two points for current injection and two points for potential measurement. For this purpose a sensitive *OM22* micro-ohmmeter is used. This apparatus offers the possibility for temperature compensation and automatic data collection.

The compression bench is shown in Figure 2; it uses a clamping force and its development was inspired by some publications [2,5,10]. Compression of the wires is achieved by turning the crank wheel at the top of the device as shown in Figure 2b; measurement of the compression force is possible for two intersecting strands. The measurement configuration (the force range of 0.5 N to 500 N) makes it possible to take several measurements on the same pair of samples. For this study we have 200 samples extracted from a copper coil manufactured by Nexans, from which we cut samples 40 cm in length in order to fit the test bench. The system for fixing the wire is shown in Figure 2c. The conductor (6) is placed on an insulating support (2) and held in position by insulating pressing blocks (3) and (4). The whole assembly is fixed to the frame (1) using two screws (5). The conductor needs to have a shape as shown in Figure 2c.

Once the mechanical contact is made between the two copper wires at a certain compression force, a pulsed current is injected on two opposite ends of the two wires. The current will flow from one wire to the other through the contact surface because of the difference of potential set between the two wires. The increase of the contact pressure will change the characteristics of the contact zone. Several sources of the measurement system error such as the external environment can affect the accuracy of low resistance measurements. To reduce these errors, the resistance measurements are made at room temperature using the Kelvin method [11]. The current circuit and the potential circuit are separated to eliminate the resistance of the connecting cables. The tests are performed with temperature compensation using sensitive thermocouple. A series of measurements according to the applied force and crossing angle are made. The contact force range is set from 0.5 N to 500 N with a contact angle variation from 0° to 90° under a pulsed current of 10 A with a time period of 0.5 s. The use of a pulsed current is to reduce the Joule heating in the contact interface. The contact force is measured using a tensile compression S-shaped type SM5424 strain gauge sensor, connected to a SM18 USB2 data acquisition type converter. Some of the parameters related to the conditions of measurements may be a source of measurement error. One of such cases arises when the angle between the conductors is 0°, as it is easy to overlook a slight misalignment of one axis against the other, resulting in the contact area to be reduced and thus the contact resistance to be overestimated.

### 2.2. Presentation and Analysis of Results

Several series of measurements have been conducted. The first series are mainly devoted to the identification of the impact force on the electrical resistance for different arrangements of wires. The average diameter of the wires is 2.06 mm. The second series are conducted to link the different parameters to each other to identify and understand their influence on the electrical contact resistance.

#### 2.2.1. Influence of the Compression Force

**Figure 3.**
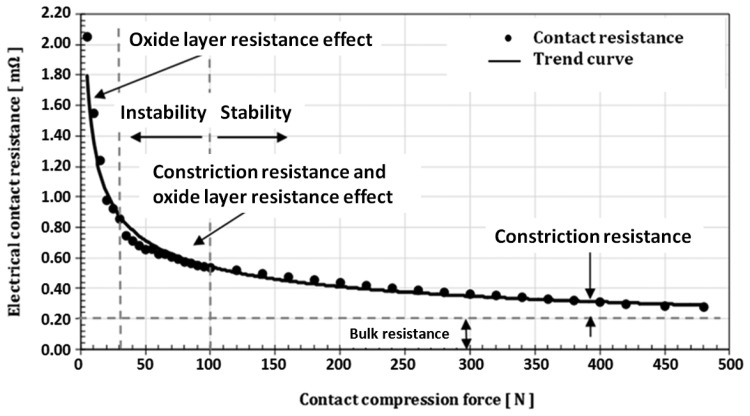
Electrical contact resistance as a function of the compression force (crossing angle of 90°) for copper (measuring current of 10 A, rated voltage drop of 20 mV, the diameter of wire 2.06 mm, crossing angle 90°).

This section mainly deals with the study of the impact of the compression force on the electrical contact resistance. The measurements are performed continuously in a 9 s interval between each measurement. The number of test specimens is 200. The number of samples was important to perform statistically relevant measures for each crossing angle. The first 10 tests were conducted on a perpendicular arrangement of the conductors (crossing angle 90°). At the end of these tests, the variation of the contact resistance as a function of the force was determined (see Figure 3).

This curve represents the average contact resistance obtained from the 10 tests. After each measurement the conductor was examined under a microscope. The imprint size was verified and its surface area was calculated. For a force of less than 30 N the imprint was invisible. This proves that the deformation was elastic. For forces greater of 30 N the imprint is visible, which means that we are dealing with plastic deformation. It may be observed that for low contact compression force (<30 N) we are dealing with elastic deformation and the current flow at the contact interface is controlled by the oxide layer, therefore the resultant contact area is extremely small. While, from 30 N to 100 N there is plastic deformation and a constriction resistance will appear in few contact points. In this zone, it may be observed that the oxide layer resistance and constriction resistance will coexist [12,13]. Finally, the stability of contact resistance can be observed up a compression force 100 N. (Table 1)

#### 2.2.2. Influence of the Force Depending on the Angle of Intersection of Conductors

**Figure 4.**
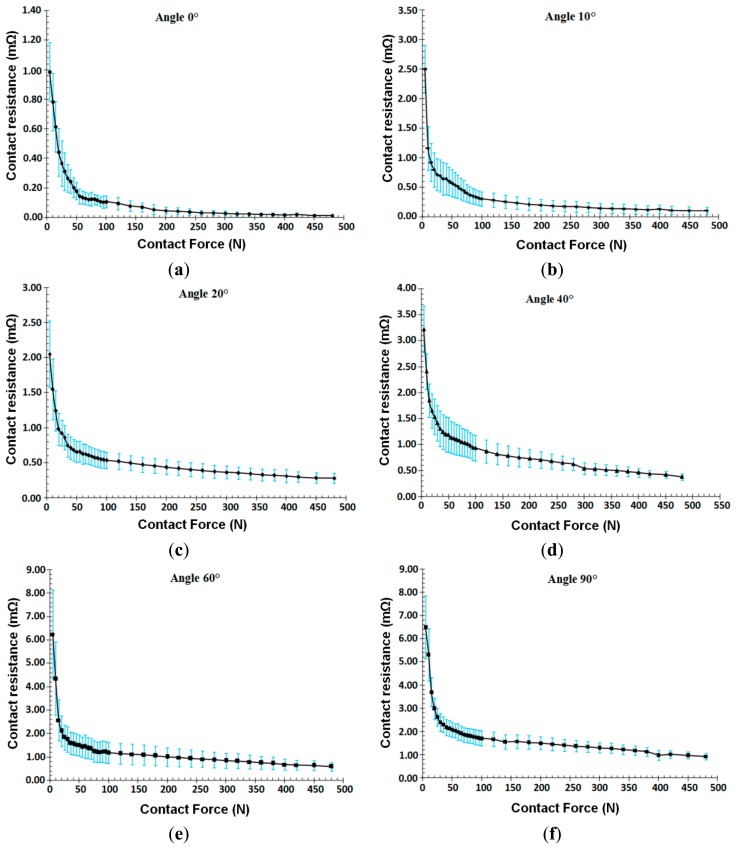
Resistance of the contact area according to the angle between the crossed wires, diameter of wire 1.8 mm.

The study of the influence of the crossing angle is made at 10 angles. The studies were conducted to investigate the reproducibility of the curves and are presented in the following only for the curves for 10°, 20°, 40°, 50°, 60°, 80° and 90°. These curves are shown in Figure 4.

For reasons of clarity, the results for some angles have not been presented. It can be seen that the dependence of the electrical resistance behaviour on the applied force is the same as identified above, and, for all specified angles. Figure 4 shows the effect of the crossing angle on the evolution of the electrical resistance. As the angle of intersection of the conductors in contact tends to a parallel arrangement of the conductors, something that occurs at the 0° angle, the more the resistance decreases and becomes more stable.

Summarising the results it can be observed that there are certain tendencies common to different measurements. In the initial part of the characteristic curves, for forces between 5 N and 30 N, there is an abrupt reduction of the contact resistance, followed by an ‘inverted knee point’ for forces in the range between 30 N and 100 N; this may be explained by a phenomenon of plastic deformation of the contact region while the rest of the material enjoys elastoplastic deformation. The last section of the curve clearly describes a fairly ‘stable’ behaviour up to the maximum force. Each of the above sections may also be related to the manufacturing process of multi-strands conductors where different forces are used when stranding the conductor. Generally, higher force the lower the contact resistance. At Figure 4 on each curve the dispersion of the measurement and the average values are shown.

**Figure 5.**
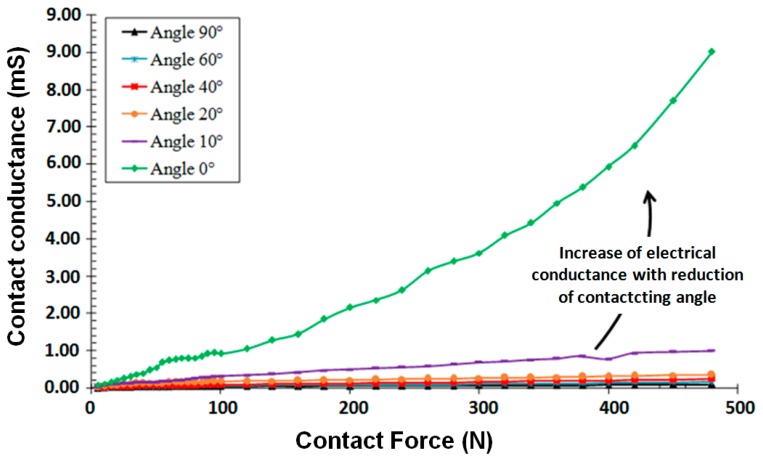
Variation of the electric conductance as a function of contact force to two extreme arrangements of conductors (angle 0° and 90°).

Figure 5 compares the electrical conductance of contact (1/RC) for the two extreme angles 0° and 90° depending on the applied force applied. As can be seen, electrical conductance is significantly higher for the cross angle 0°. The reason for this is an increase of the contact area with the reduction in angle of intersection of the conductors.

### 2.3. Influence of Variation of the Contact Area

**Figure 6.**
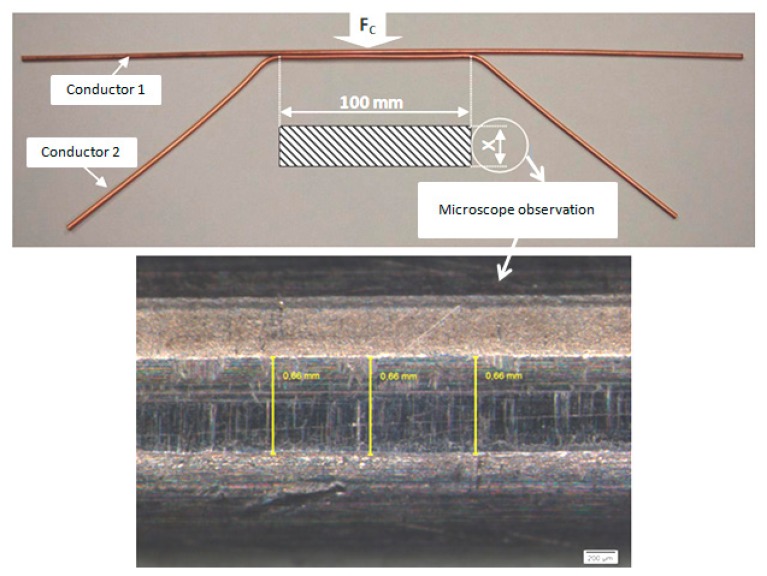
Measurement of the imprint at a crossing angle 0° for a contacting force of 480 N. and a stress of 7.27 N/mm^2^.

**Figure 7.**
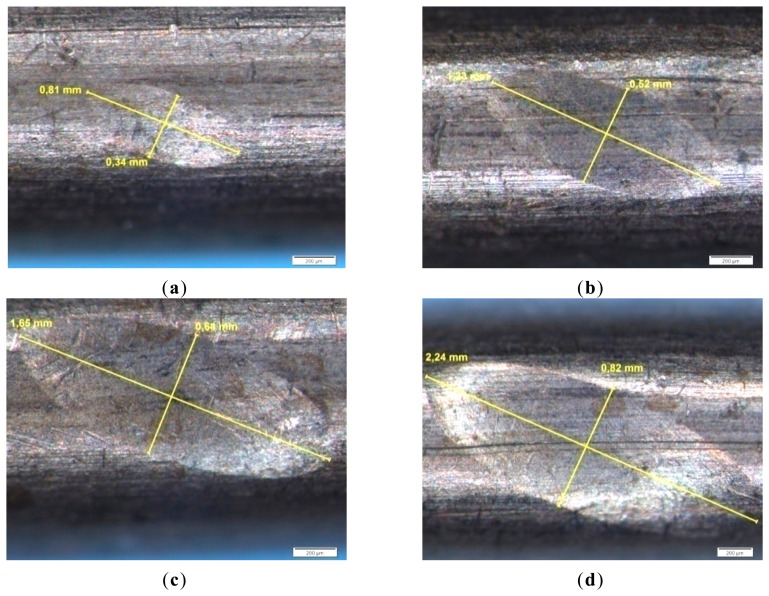
Residual imprints on the copper wire for a contacting angle of 45° under ascending contact forces. (**a**) F_C_ = 100 N, averaged stress =115 N/mm^2^; (**b**) F_C_ = 200 N, averaged stress = 99.5 N/mm^2^; (**c**) F_C_ = 300 N, averaged stress = 90.4 N/mm^2^; (**d**) F_C_ = 480 N, averaged stress =83.2 N/mm^2^.

Another effect also depending on the applied load is observed on changes in the contact resistance during testing. This effect relates to the contact area formed by the crossing angle between the two wires. It should be noted that not only is the distribution of the contact resistance non-uniform, but the depth of the penetration of one conductor into another is not constant. In order to incorporate this into our model, the mechanical analysis needs to be coupled with the electrical description as when two conductors penetrate each other their physical and mechanical properties changed. Accurate dimensions of the imprint were established under the microscope (vertical and lateral resolutions equal to 200 μm) and are shown in Figures 6 and 7. Figure 6 shows the parallel disposition of the conductors. The area of the contact surface under the maximum force at the angle of 0° may be found analytically by approximating the imprint between two parallel cylinders by a rectangle of known length equal to the length of the sample used in the measurements. Applying a large force of 480 N, results in smoothing out the unevenness of the contact surface and thus increasing the contact surface area up to the full area of the relevant rectangle, as shown in Figure 6. The rectangular area is then calculated as: S = 100 × [(0.386 + 0.258 + 0.279 + 0.274)/4] = 2.35 mm^2^.

Another example of imprints measurements for a crossing angle of 45° are shown for different contact stresses in Figure 7. The imprints are always elliptical at all angles, except for 0° (parallel conductors). When the angle increases the ‘width’ of the imprint is reduced while the ‘length’ remains constant.

**Figure 8.**
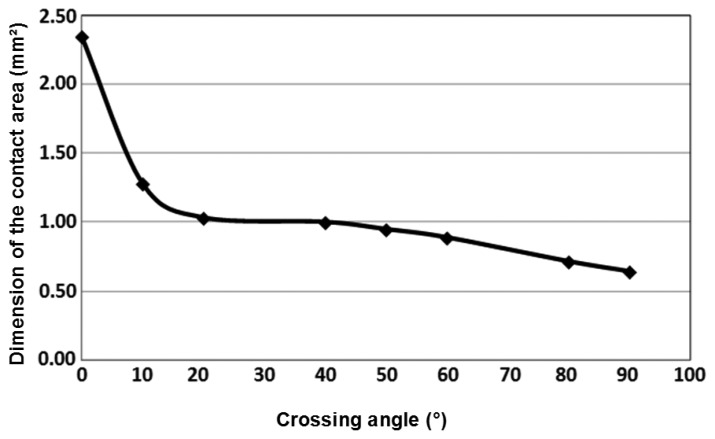
Dimensions of the contact zone as a function of crossing angle (compressive force 480 N).

**Figure 9.**
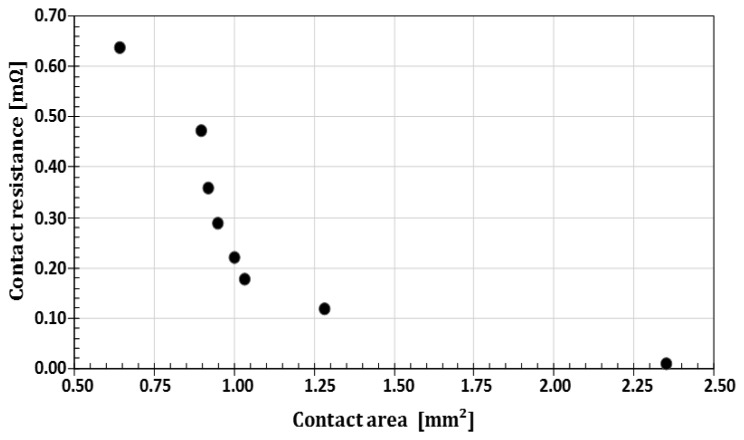
Contact resistances as a function of the areas' dimensions.

The uniform distribution of the stress on the contact area was established at this stage. The non-uniform stress repartition was taken into account during electro-mechanical simulations. From these experimental tests, we observe an increase of the dimensions of contact zone between the wires when the crossing angle is decreased (Figure 8). The contact resistance will then decrease to become stable for parallel wires as shown in Figure 9. In the case of stranded conductors, of the type supplied by Nexans and studied here, the stranding lay length determines the angle at which conductors intersect. Figure 8 and 9 show the resulting contact resistance and contact area as a function of the angle of intersection. Significant influence of this angle on the value of the resistance may be observed. The contact area shrinks as the angle increases; for example, the difference between the contact area at the angles of 0° and 90° is 1.491 mm^2^.

A change in the contact area affects the current distribution. As the angle of intersection is related to the lay length with which the conductor is stranded, it is clear that we can influence the resultant resistance of the conductor by changing the lay length ratio.

## 3. Numerical Model

### 3.1. The Design of the Conductors

**Figure 10.**
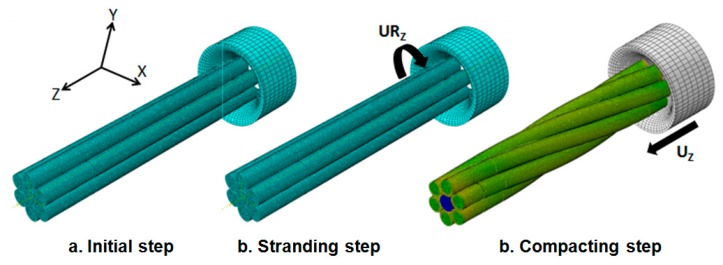
Abaqus stranding and compacting process boundary conditions.

The stranding and compacting process is an operation that combines two simultaneous displacements: translation and rotation. The wires are subjected to three forces: traction, torsion and compression, as shown in Figure 10. The traction force is due to the linear speed provided by the winch, the torsion is generated from the yokes and the compression force from the area reduction at the lay point. The mechanical simulations have been performed to determine the actual deformed geometry of the conductor, the residual stresses, the plastic strain and material hardening due to area reduction of the strands. The results were linked to experimental measurements to quantify the changes on material electrical conductivity. These simulations were developed using the Abaqus program's explicit dynamic module. This module offers the possibility to analyse complex physical problems with large nonlinearities, complex geometry shapes and interface contact conditions. The total resistance of the stranded conductor depends on a number of parameters. The main parameters are the conductivity of the material and the overall design of the conductor (the diameter of the wires, the number of wires, the number of layers, the lay lengths of each layer, the direction of the layers, the compaction of conductors) and the contacts between the wires. Different stages of the production of the cable are sources of factors that may affect the electrical resistance. Indeed, the deformations due to the stranding process, the contact pressures between the wires, the reduction of area due to pulling forces, *etc.* strongly impact the electrical behaviour of the material. To facilitate identification of contact surfaces and definition of relevant equivalent volumes by Abaqus a procedure that can reproduce the twisting process was prepared,. This procedure is the following: first the geometry with a parallel beam of wires is prepared, secondly tensional forces are applied. The next step is a compaction and extrusion of the conductor. This process is shown in Figure 10.

The electrical phenomenon in the contact area between strands is complex; this interface is controlled by the contact pressure and the material's ability to undergo plastic deformation. It was determined that real surfaces are not flat, but have many bumps [10,14–17]. The current distribution in the cable depends mainly on the strength of each strand and the quality of the electrical contact between the strands. When the peripheral strands are subjected to tensile stress inherent in the manufacture of the conductor, the electrical resistance tends to increase slightly; the current lines will focus around the strands having the smallest resistance. Even small levels of mechanical stress, induce differences between the strands, that result in imbalances between the wires connected in parallel. Small imbalances can result in significant differences between local current densities. It is also to be considered that during crushing the contact zone changes its properties.

### 3.2. FEM Modelling of the Stranding Process with the Abaqus Explicit Modulus

**Figure 11.**
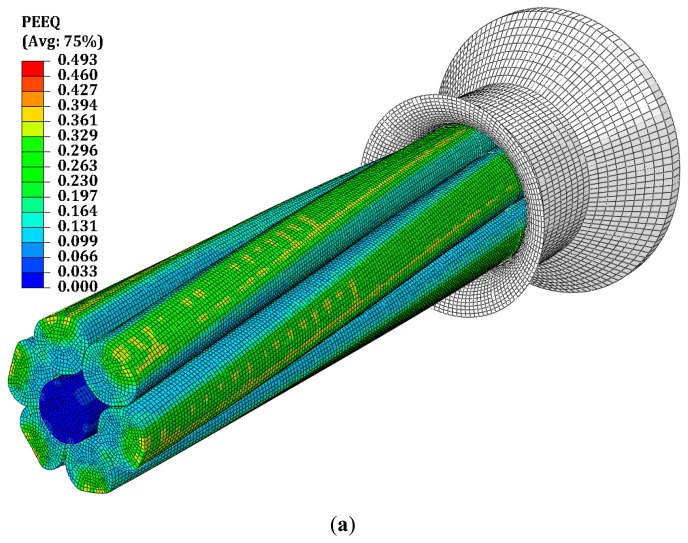

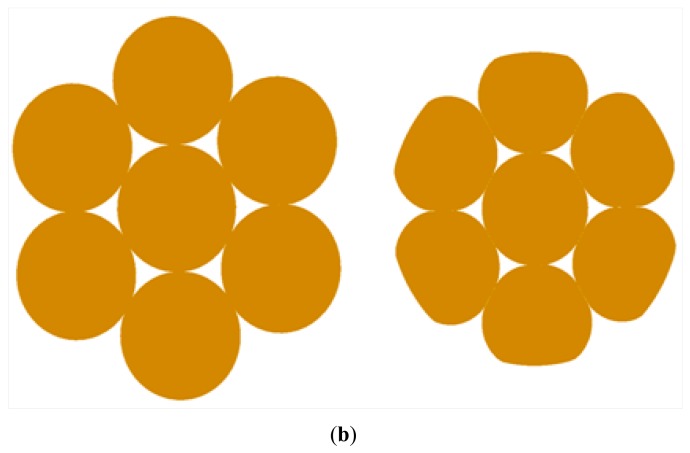
Simulation of the compacting process: (**a**) Equivalent plastic deformation; (**b**) conductor cross-section before and after the stranding and compacting process.

The plastic deformations during the compacting process introduce local dislocations. Through extensive simulations and supporting measurements, conductor resistance data has been established for various conductor design parameters. The main objectives of the mechanical simulations consist of determining the actual deformed geometry of the conductor, the residual stresses, the plastic strain and the hardening range due to the area reduction. In fact, the mechanical results linked to the electrical experimental measurements will allow us to quantify the changes of the material electrical characteristics as the specific electrical resistivity. In this study we use a penalty contact algorithm with finite sliding. The advantage of a finite sliding contact is that all the rotations and translations are authorized in the contact interfaces. The simulations are calculated under quasi-static loadings. The generated mechanical stresses and plastic deformations are shows in Figure 11.

**Figure 12.**
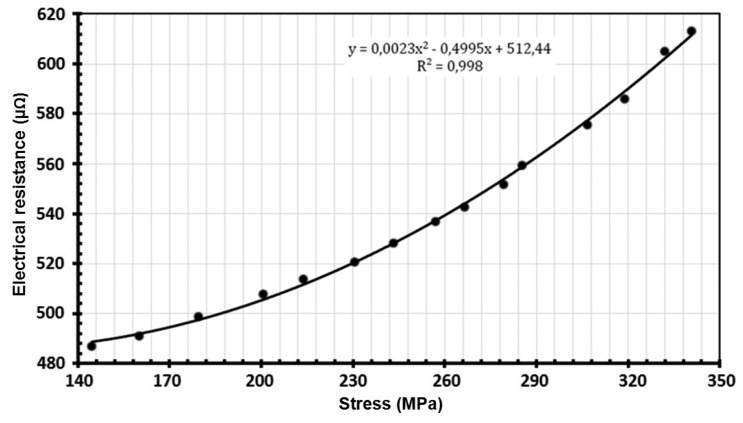
Electrical resistance in function of mechanical stress.

The relationship between the mechanical stress and copper electrical resistivity has been established. The curves shown in Figure 12 have been determined by a cold drawing process. The equivalent conductor hardening caused by compaction determined by the mechanical simulations were linked to the measured electrical resistivity of copper. After compaction, the equivalent resistivity of copper has been increased by 0.4% (1.71038E−08 Ω·m^2^/m) from the initial copper resistivity (1.70382E−08 Ω·m^2^/m).

### 3.3. Electrical Model

**Figure 13.**
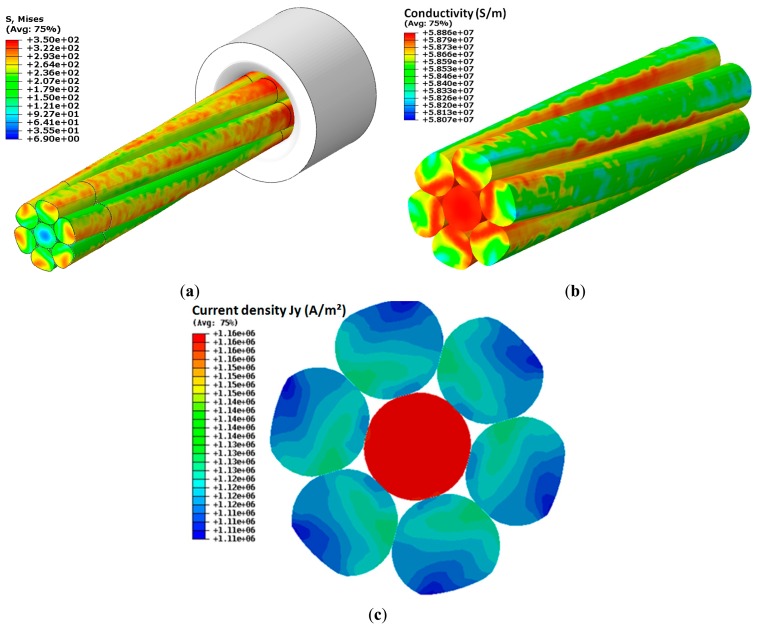
Distribution of residual stress (**a**); the resulting relative conductivity distribution (referred to the basic conductivity) (**b**) and resulting current density (**c**).

To implement out the electrical model we use the Abaqus direct current flow module. The deformed geometry has been re-meshed. A difference of electrical potential is given between the two ends of the conductor. At the input surface we defined the ground U = 0 mV, and at the output surface a voltage equal to U = 1 mV. The geometry has been meshed with tetrahedral elements of 0.2 mm. The length considered for the DC model is 50 mm. The temperature is fixed at 20 °C. The behaviour law of the contact resistance as a function of contact pressure and crossing angle was implemented in Abaqus as an interpolated function. Based on the distribution of stresses in the conductor, the distribution of the conductivity and current density were calculated. Figure 13 shows the results of distribution of stresses at the cable cross-section and the resulting spatial distribution of electrical conductivity. The copper conductivity of the compacted wires has been decreased by 2%, but even so a small change can significantly affect the distribution of current density and resultant resistance of the cable.

The electromechanical model was developed to understand and predict the influence of the stranding and compacting process on mechanical and electrical characteristics of a concentric conductor (1 + 6). Several mechanical and electrical simulations have been performed in order to quantify the influence of the area affected by mechanical deformation and hardening on the conductors' electromechanical behaviour. To validate this model, we compared the results obtained by experimental measurement and finite element simulations. Two main parameters are chosen to validate the numerical model: the weight and the electrical resistance of the conductor per kilometre. The differences between the two models has been identified, as shown in Table 2.

A comparison of measurement and simulation of an ideal transition zone indicates the existence of transition layers of different conductivity than the conductivity of copper. It has been shown that the voltage and current distribution depend strongly on the force used during the conductor manufacturing. Another problem is that during the crushing, the properties of the contact zone change. We have shown how important the contact zone is and how it changes when compacting forces are applied. The current density distribution is also heavily influenced by the structure of the contact regions between the wires and the resistance of the transient layer.

## 4. Conclusions

In this paper a new method has been presented to predict mechanical and electrical behaviour of a concentric conductor (1 + 6) according to the stranding and compacting process parameters. The original objective of this work was to consider the mechanical deformations of the conductor and their impact on material electrical conductivity. A relationship between copper electrical conductivity and stresses has been characterized by experimental measurements.

The condition of the conductor surface is very important and affects the reliability and repeatability of the measurements. Hundreds of samples were tested and results averaged to make them meaningful, so that relationships could be established showing the dependence of the contact resistance between stranded conductors on the acting compression force.

The experimental results show that the range of the electrical resistance is dependent of three major parameters: the compression force to which the conductor is subjected, the size of the two contact surfaces, which are directly related to the applied force, and the intersection angle between the strands of the cable. The resulting characteristics of contact resistance and conductivity of the single wire *versus* strain are used for modelling a real 1+6 cable wire system.

## Figures and Tables

**Table 1.** Measurement of the electrical contact resistance (mΩ) as a function of the applied force (N) and the dimension of the contact area (mm^2^) measured for crossing angles 90°, 60° and 45°, for the wire of 1.80 mm diameter.

**Crossing Angle**	**Measured Average Force [N]**	**Contact Area [mm^2^]**	**Contact Resistance [mΩ]**
**90°**	50.58	-	3.878
	101.50	0.221	2.328
	202.14	0.478	1.500
	300.75	0.724	0.984
	401.37	1.021	0.785
	481.12	1,287	0.684

**60°**	50.58	//	2.854
	101.50	1.027	1.703
	202.14	1.979	0.945
	300.75	2.859	0.688
	401.37	4.134	0.479
	481.12	4,971	0.413

**45°**	50,58	//	2.242
	101.50	0.865	1.587
	202.14	2.009	0.814
	300.75	3.318	0.561
	401.37	4.366	0.309
	481.12	5.770	0.276


**Table 2.** Main results of actual and FEM models electrical resistance and conductor weight prediction.

**Design Parameters**	**Actual Manufactured Model**	**FEM Model**
Conductor weight [Kg/km]	218.03	218.00
Electrical resistance [Ω/km]	0.707	0.7086
Resistance average gap [%]	0.23%	
